# Qigong Exercise Balances Oxygen Supply and Acid-Base to Modulate Hypoxia: A Perspective Platform toward Preemptive Health & Medicine

**DOI:** 10.3390/medsci11010021

**Published:** 2023-02-28

**Authors:** Junjie Zhang, Qingning Su, Shengwen Calvin Li

**Affiliations:** 1 School of Physical Training and Physical Therapy, Shenzhen University, Shenzhen 518060, Guangdong, China; 2 Center of Bioengineering, School of Medicine, Shenzhen University, Shenzhen 518060, Guangdong, China; 3 Neuro-Oncology and Stem Cell Research Laboratory (NSCL), CHOC Children’s Research Institute (CCRI), Children’s Hospital of Orange County (CHOC), 1201 W. La Veta Ave., Orange, CA 92868-3874, USA; 4 Department of Neurology, School of Medicine, University of California-Irvine (UCI), 200 S Manchester Ave Ste 206, Orange, CA 92868, USA

**Keywords:** eastern medicine, Qigong, hypoxia, acid-base balance, cancer, metabolism

## Abstract

Qigong is a meditative movement with therapeutic effects and is commonly practiced in Eastern medicine. A growing body of evidence validates its health benefits, leading to mechanistic questions about how it works. We propose a novel mechanism by which the “acid” caused by hypoxia affects metabolism, and the way it is neutralized through Qigong practice involves the body’s blood flow and vasculature modifications. Specifically, Qigong exercise generates an oxygen supply and acid-base balance against the hypoxic effects of underlying pathological conditions. We also propose that Qigong exercise mediated and focused on the local hypoxia environment of tissues might normalize the circulation of metabolic and inflammation accumulation in the tumor tissue and restore the normal metabolism of tissues and cells through calm, relaxation, and extreme Zen-style breathing that gravitates toward preemptive health and medicine. Thus, we propose the mechanisms of action related to Qigong, intending to unify Eastern and Western exercise theory.

## 1. Introduction

By lowering negative symptoms and mood disorders and boosting function, alternative exercise traditions (AETs) like Pilates, yoga, Tai Chi Chuan, Qigong, and various kinds of dance have the potential to enhance a variety of outcomes for cancer (breast cancer [1]; breast cancer and weight loss [2]) survivors, which attracts much attention recently, particularly for patients with complex underlying conditions like type 2 diabetes, cardiovascular disease (CVD), and cancer [3]. Qigong, as a Meditative Movement for breathing, body movement, meditation, and awareness, came from Eastern medicine, the practical, philosophical, and psychological background of Ki (in Japanese) or Qi (in Chinese) [4]. Qigong exists in various forms of Complementary and Alternative Medicine (CAM), including movements of (1) ‘Plucking the Stars,’ (2) ‘Lotus Leaves Rustle in the Wind,’ ‘Chaoyi Fanhuan Qigong (CFQ)’ [5], (3) ‘Baduanjin exercise’ [6], (4) ‘Tai Chi’ [7], (5) ‘Pacing Forwards and Backwards,’ and (6) ‘Zhan Zhuang Qigong’ [8]. All Qigong forms are thought to benefit meridians, flexibility, strength, articular stimulation, neuro-integration, respiratory effect, fascial stretch, visceral massage, balance challenge, CranioSacral pump, lymphatic and venous return, and glandular stimulation [9]. Qigong research has gravitated toward clinical outcomes, e.g., blood glucose, depression, and anxiety among patients with type II diabetes and emotional disorders [10]; however, little is known in its mechanism at the level of physiology, cognitive function, and therapeutic pharmacology.

Qigong includes a wide range of contents, characterized by the combination of mind, Qi, and body exercise through the practitioners’ subjective efforts, including the contents of body adjustment, heartbeat adjustment, breathing adjustment, self-massage, and other body activities. Qigong is a self-mental and physical exercise that, through the way of self-suggestion, drives the consciousness to enter the state of self-quietness, and through benign psychological adjustment and unique breathing styles such as abdominal breathing to balance the physiological functions among different systems or tissues in the body even to achieve the purpose of preventing and treating diseases.

The practice of Tao (Qigong) aims at, first of all, maintaining health and dispelling disease, and secondly, prolonging life. Training in respiration is an important content of Taoist practice. The main feature of this type of training is to inhale or exhale as you expire and inspire. Taoism believes that through this way of breathing, we can maximize the acquirement of energy from the environment and promote the body’s inner potential. Physiologically, this practice can be considered supercharging breathing to improve lung cells’ air exchange capacity and elevate red blood cells’ oxygen loading to increase oxygen supply in normal metabolism and physiological function. This breathing method promotes the microcirculation system of the whole body from the conditional state of anxiety-induced cold hands and an anxiety-induced heart rate to the state of warm fingers and a calm heart. It accelerates the transport of local metabolites (e.g., inflammation molecules) to avoid accumulation. At the same time, the body energy movement (Qi) mediated by oxygen and nutrients are adequately circulated for the whole body, through which the inflammation molecules of local tissues in the abnormal state can be relieved, a mechanism by which Qigong acts as a therapeutic measure in the disease state. Such positive biofeedback vegetative pathways might be activated to correct the dysfunctional patterns in critical stressful situations of disease [11]. Taoists believe that Qigong can enhance the function of the respiratory system organs, accelerate the body’s metabolism, and stimulate the physiological potential to improve the body’s resistance to disease and self-repair ability through meditative, energetic, and physical pathways.

Here, we will expound on the effect of Qigong in the recovery process after exercise or damage caused by lack of oxygen, ischemia, hypoxia, and the effect of Qigong in disease-dispelling from the angle of oxygen on energy metabolism, metabolites, and acid-base balance. We propose a novel mechanism by which the “acid” caused by hypoxia affects metabolism and how the Qigong body-mind link neutralizes it.

## 2. Effects of Qigong on Energy Metabolism and Metabolite Accumulation in Post-Exercise and Hypoxic Tissues

**Figure 1:**
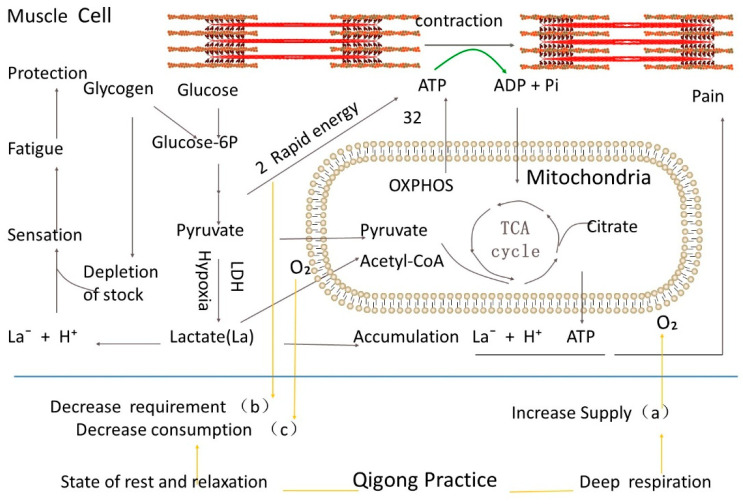
Schematic representation of glucose metabolism during exercise. The orange lines represent the state of Qigong practice. The blue line presents the cell membrane. Muscle contraction requires the hydrolysis of ATP, a form of chemical energy, for the actin-myosin cross-bridge cycle. Glucose and glycogen are the forms of energy stores. Glucose in the cells is phosphorylated into glucose-6-phosphate (glucose-6P). Glucose-6P is then metabolized through glycolysis to produce two pyruvate molecules and two rapid-energy ATP molecules. Two pyruvate molecules can enter mitochondria, which are metabolized through the tricarboxylic acid (TCA) cycle and oxidative phosphorylation to produce 32 more ATP molecules when oxygen is available. Pyruvate can otherwise be reduced into lactate-by-lactate dehydrogenase (LDH) when the cells are under the hypoxia condition, and a process is called glycolysis. So the complete oxidation of glucose produces much more molecules of ATP in the mitochondria (32 ATP) than in glycolysis (2 ATP). The slow ATP production in mitochondria and shortage of oxygen supply during exercise drives glucose metabolism through glycolysis for rapid energy. Under hypoxia, the metabolite pyruvate is converted into lactate by LDH. The low efficiency of ATP production in glycolysis results in the rapid depletion of energy stock glycogen and lactate accumulation. Lactate accumulation and its existence as La^−^ and H^+^ play a role in fatigue sensation for tissue protection and the signal of tissue disorders such as pain. Through training deep respiration and keeping our body in a state of relaxation, Qigong practice improves the hypoxia state of the tissue in three ways: (a) by Increasing the supply of oxygen; (b) by decreasing the requirement of rapid energy; (c) decreasing the consumption of oxygen.

Muscle contractions produce force and heat, which looks like mechanical energy but consumes chemical energy from ATP. When we exercise, our muscle cells contract and undergo a series of metabolic activities that produce various metabolites and heat. Fatigue and muscle soreness are the most common symptoms after strenuous exercise. The intracellular lactic acid accumulation is a disadvantage during muscle activity [12], thereby decreasing pH that is believed to be involved in muscle fatigue, which results from ionic changes on the action potential, extracellular and intracellular ions, and many intracellular metabolites, failure of sarcoplasmic reticulum (SR) Ca2+ release by various mechanisms, and the effects of reactive oxygen species [13]. No matter what exercise we do, we end up feeling tired or exhausted. The accumulation of metabolites protects our bodies from harm by the perception of tiredness, which can reduce physical activity or stop performance. During intense exercise, ATP is mainly generated from blood glucose and muscle glycogen to fuel glycolysis in muscle cells; nonetheless, the lactate paradigm has changed and now recognizes lactate as a vital metabolic intermediary, a specific transportable fuel for aerobic metabolism, and maybe a mediator of redox state between various compartments both inside and between cells [14]. Extensive exercise consumes oxygen as well as energy quickly. Therefore, our bodies need to obtain energy and reduce oxygen consumption as soon as possible, which drives the energy metabolism of cells to glycolysis for rapid energy ATP and less oxygen consumption (Figure 1). Through glycolysis for quick energy production, two molecules of ATP can be produced for each glucose molecule. The metabolite pyruvate is further catalyzed by pyruvate dehydrogenase to form lactate under hypoxia conditions. Under normal conditions, pyruvate is transferred to mitochondria where 32 molecules of ATP are eventually produced through a process called oxidative phosphorylation for neuroenergetics of integrated cooperativity between astrocytes and neurons [15]. Compared to oxidative phosphorylation, glycolysis is obviously a less productive pathway and will deplete the cells’ energy store glycogen sooner. Although lactate and pyruvate can be transferred to mitochondria for more energy production under normal circumstances, the need for rapid energy and insufficient oxygen supply during intensive exercise drives the glucose metabolism in muscle tissue from normal aerobic respiration to glycolysis, resulting in the accumulation of lactate.

**Figure 2:**
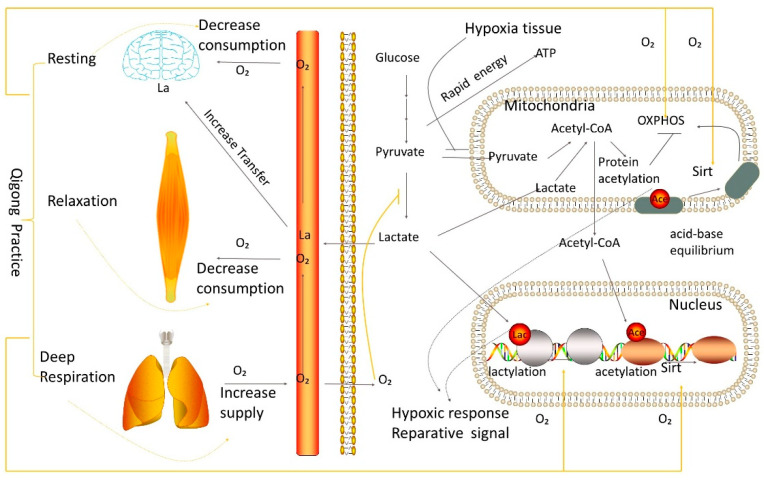
Schematic representation of metabolic oxygen level, lactate, and acid-base equilibrium in brain, muscle, lung, blood, heart, and liver, as modulated by Qigong practice. The orange lines represent the state of Qigong practice. The final metabolic step in the synthesis of adenosine triphosphate is the mitochondrial oxidative phosphorylation (OXPHOS) system (ATP). The genes for the five multiprotein complexes that comprise the OXPHOS system, Complex I through Complex V, are encoded by the mitochondrial (mtDNA) or nuclear genomes (nDNA). For effective OXPHOS activity, close to 300 proteins are required, and O_2_ controls OXPHOS activity. The metabolic pathway known as OXPHOS, also known as electron transport-linked phosphorylation or terminal oxidation, is where cells use enzymes to oxidize nutrients to release chemical energy and produce 30 to 32 ATP molecules. This OXPHOS action contrasts glycolysis, an independent form of OXPHOS, and only has 2 ATP molecules. The OXPHOS, which generates roughly 90% of the ATP molecules produced in the brain, drives neuronal activity processes such as pre- and postsynaptic action potentials, neurotransmitter release, and postsynaptic currents. Substrate feedback inhibition, protein stability, mtDNA and nDNA transcriptional regulation, translational effects via RNA levels, and post-translational changes such as phosphorylation, acetylation, and lactylation tightly regulate OXPHOS.

Lactate (La) generally exists in the form of La^−^ and H^+^ ions (Figure 2). The accumulation of La^−^ and the acidic environment caused by H^+^ put local tissues in an abnormal state. After a normal oxygen supply is restored, the glucose metabolism again enters the tricarboxylic acid aerobic respiration cycle and produces more ATP. Pain may occur if the accumulation of lactate in an acidic environment is not effectively transferred [16] (Figure 1), as shown in a non-hydrolyzable form of ATP (α,β-meATP) alone and combination with lactate and acidic pH.

Under normal circumstances, the human brain consumes 20% of the body’s total energy requirement with slightly more than 2% of adult body weight [17]. Qigong exercise’s resting and relaxing state effectively reduces oxygen consumption in significant oxygen-consuming tissues such as the brain and muscles to relieve the body’s hypoxia (Figure 1). The cells resume aerobic respiration, and glucose metabolism returns to the high-energy, productive pathway in mitochondria through oxidative phosphorylation. Resting and relaxing states reduce the body’s energy requirement and the need for rapid energy replenishment. Lactate, the final product of glycolysis, can re-enter mitochondria for oxidative phosphorylation and obtain more ATP. Deep breathing during the Qigong exercise maximizes oxygen exchange in the lungs, increasing blood oxygen levels and ensuring the need for oxygen in tissues throughout the body. Therefore, Qigong exercise provides oxygen supply and high-productive energy metabolism in tissues by reducing oxygen consumption, reducing the need for rapid energy replenishment, and increasing blood oxygen levels. Therefore, Qigong exercise can quickly rejuvenate anoxic tissues and reduce fatigue or damage caused by harmful substances or tissue acidification.

Other supporting evidence came from a recent study. Worldwide, ischemic disorders, including myocardial infarction and stroke, are the leading causes of death. To date, therapies intended to stop ischemia damage or restore functioning tissue have not proved effective. In cardiac, cerebral, renal, and retinal models of ischemia, kynurenic acid (KynA), a product of tryptophan metabolism, is tissue protective; however, the mechanism behind this protection was unknown [18]. When imported, KynA indirectly interacts with adenosine triphosphate (ATP) synthase inhibitory factor 1 (IF1) to reduce ATP depletion during ischemia by encouraging the dimerization and deactivation of ATP synthase. This action activates downstream signaling, as speculated in the report. However, when treated with KynA, mouse hearts lacking GPR35 lost the therapeutic impact. Neonatal cardiomyocytes activated with KynA and GPR35 linked to mitochondria appeared to interact indirectly with ATP synthase inhibitory factor subunit 1, lowering ATP loss during ischemia. The results provide more evidence of the potential therapeutic use of GPR35 agonists to lessen the negative consequences of ischemia injury. [19]. How Qigong might play preventive roles in reducing ischemic damage remains to be elucidated for the linked action of GPR35.

## 3. Qigong Exercise Balances the Whole Body by Accelerating Material Circulation, Balancing Acid-and-Base in Time, and Coordinating Metabolism and Gene Regulation

Lactate level is known to increase when tissue cells are starved for energy and require more oxygen than they can supply, often during extensive exercise and ischemia [20] (Figure 2). Under the condition of hypoxia, glucose catabolism is mainly carried out through the inefficient energy metabolism pathway glycolysis to produce a large amount of lactate, a.k.a., the Warburg effect, one of the hallmarks of tumors, which makes large amounts of lactate and generates an acidic tumor microenvironment [21]. These acidic products must be balanced by transport or conversion to other products. Otherwise, these lactic acidic products might promote tumor initiation and progression [22]. From the perspective of oxygen supply, Qigong breathing can significantly relieve the local hypoxia state of the human body. During Qigong practice, the whole body often feels a sense of expansion. At this time, the tissue microcirculation is filling, and the local hypoxia state will be relieved.

As the microcirculation is improved, the metabolite accumulation in local tissue will be quickly transferred to other places, reducing the local pressure of metabolic accumulation. More importantly, burdensome specific metabolites (e.g., lactate) in one tissue may be important regulators in another organ, as lactate acts as a mediator of the beneficial effects of exercise on brain health. Lactate plays a dual role as an energy supply substrate and a signaling molecule in this metabolic coupling process [23]. This metabolic coupling process manifests. “On the other hand, lactate acts as a signaling molecule to activate downstream signaling transduction pathways by specific receptors, inducing the expression of immediate early genes and cerebral angiogenesis. Moderate to high-intensity exercise not only increases lactate production and accumulation in muscle and blood but also promotes the uptake of skeletal muscle-derived lactate by the brain and enhances aerobic glycolysis to increase brain-derived lactate production. Furthermore, exercise regulates the expression or activity of transporters and enzymes involved in the astrocyte-neuron lactate shuttle to maintain the efficiency of this process; exercise also activates lactate receptor HCAR1, thus affecting brain plasticity”.

Lactate, therefore, is the end product of glycolysis. Its role has gradually changed from metabolic waste to energy substrate for neural activity and important intercellular signaling molecules in the brain [24]. Lactate is a burden in hard-working muscles or oxygen-starved tissues, but it is a necessary fuel and regulator in the brain [15]. Thus, previously harmful metabolites locally will be balanced globally as long as the metabolites are transferred promptly and efficiently (Figure 2). The differential regulation resolves this seeming dilemma and makes it clear. The prevalence of each pathway depends on the potency of the glutamatergic stimulation. L-Lactate is implicated in two unique and independent processes: an NMDAR-mediated potentiation pathway (or NADH pathway) and a neuroprotective pathway (or Pyruvate/ATP pathway). [25]. When the human body is in the state of Qigong exercise, the blood hemoglobin oxygen content is increased. There is a feeling of expansion throughout the body during training; tissues and blood vessels are in a state of fullness.

On the one hand, this transition is conducive to transporting substances around the body to avoid nutritional deficiency and excessive accumulation of harmful substances. On the other hand, some tissues and organs which are maladjusted due to ischemia can be restored to their normal functions in time (Figure 2). So, it is no surprise that Qigong exercise may accelerate patient recovery and has a more significant clinical effect in patients with COVID-19 than standard therapies alone, e.g., Baduanjin exercise, one form of Qigong for rehabilitation after COVID-19 [26]. The mechanism by which Qigong exercises consciously manipulate breathing likely achieves lung-related physiology and clear inflammation molecules for recovery after COVID-19 [27], as shown in Figure 2. This action was efficacious for elderly patients [28] for improving cognitive functions via an interleukin-6-hippocampus pathway based on a randomized active-controlled trial [29]. Most Qigong studies of COVID patients acted as an adjuvant to other treatments. The major challenge is to tear out the specific contribution of the Qigong exercise in these studies to identify the mechanisms of Qigong-specific impacts in drug-drug interactions, particularly in human COVID conditions.

Due to a reduction in energy metabolism and the formation of hazardous reactive oxygen species (ROS) brought on by shocks to the numerous defective enzymes, inefficient OXPHOS through complexes I–IV causes human diseases, also known as OXPHOS sickness (particularly complexes I, II, and III). Another factor contributing to mitochondrial dysfunction is complex V defects. OXPHOS disorders can result from mutations in both nuclear and mitochondrial genes. The most prevalent of these abnormalities are Complex I and Complex IV, although Complex III deficiencies are uncommon. Complex I dysfunction is believed to play a role in several neurological conditions, including schizophrenia, Parkinson’s disease, and diabetes. Paraganglioma is a kind of cancer in which complex II deficits are present. Complex IV has been linked to genetic changes in Alzheimer’s disease, and data suggest that hypoxic cancer cells have lower levels of this complex. The practice of Qigong may influence the comprehensive management of oxygen level, lactate, and acid-base equilibrium (many organs: brain, lung, muscles, heart, blood, and liver metabolism). Thus, through stillness, relaxation, and intense breathing, Qigong exercise can mediate the overall body’s physiology and the localized hypoxic environment of tissues that may normalize the circulation of metabolic and inflammatory accumulation in abnormal tissues and restore the normal metabolism of tissues and cells (refer to the text for metabolites-mediated feedback regulations in A, B, and C states with Qigong practice).

Extensive exercise or ischemia can elevate the lactate level in tissue cells by regulating the oxygen level that modulates the metabolism (Figure 2). The accumulation of lactate will result in tissue acidity and a series of changes in cell metabolism. Protein lactylation on the basic amino acid lysine is a direct and effective neutralization response. In cells, lactate, which causes tissue cell acidification, is neutralized by transferring to the basic amino acid lysine of proteins. Proteins such as histones in the nucleus are modified by lactylation, leading to epigenetic responses to hypoxia. In mitochondria, lactate mitochondria can be transformed to acetyl-CoA, by which proteins involved in oxidative phosphorylation are acetylated to temporarily stop their normal functions and reduce oxygen consumption in response to the hypoxic condition. These responses are an emergency action for the abnormal state of tissue where the accumulative metabolites must be removed as soon as possible, or a normal supply of oxygen should be resumed. Qigong exercise can provide conditions for tissues to return to normal by (A) Qigong exercise improves microcirculation and accelerates blood circulation so metabolic accumulations such as lactate can be transported to other body parts for utilization; (B), the state of relaxation during Qigong exercise can reduce the requirement of oxygen supply in the brain and muscles, two major oxygen consumption sites in the body; and (C), deep breathing during Qigong exercise can increase oxygen exchange in the lungs, increase blood oxygen concentration, and timely alleviates the local hypoxic condition of the tissues. Through these ways, glucose metabolism restores the oxidative phosphorylation pathway after the modified proteins are de-acetylated by the enzyme Sirt. The lactylated proteins in the nucleus are also returned to normal by removing modifications.

Another way to balance lactate accumulation is through lactation to the basic amino acid lysine of protein [30]. Lactate can also be metabolically converted to acetyl-CoA, balanced by acetylase transfer to lysine, the basic amino acid of protein [31]. This conversion is an obvious form of acid-base equilibrium, in which the acidic metabolite lactate is balanced by transferring to lysine, the basic amino acid of proteins. Inside the cell, the nucleus contains acidic genetic material called DNA, which is bound to a large number of basic proteins such as histones. These proteins perform functions ranging from structural function to regulation of DNA synthesis and gene expression [32]. Another DNA-containing organelle in the cell is the mitochondria, where the cellular energy metabolism process known as oxidative phosphorylation occurs. Many basic proteins, including those involved in ATP synthesis and mitochondrial fusion, are found in the mitochondria. For acid-base balance, lactylation and acetylation target the nucleus and mitochondria, the cell locations where basic proteins are concentrated in the highest amounts. The usual operation of these molecules will momentarily cease if their site of lysine is lactatylated or acetylated [31] (see Figure 2). This notion is an emergency mechanism for hypoxia.

The machinery molecules for oxidative phosphorylation receive feedback from the accumulated metabolites of hypoxia conditions and press the pause button in time. During this regulation, the acidic molecule lactate or its derivate in the cell is transferred to the basic amino acid lysine, which perfectly completes the acid-base balance in the cell (Figure 2). Restoring these molecules to their normal function requires a supply of oxygen and removing the modification called delactatylation or deacetylation catalyzed by enzymes such as deacetylase Sirt [33]. Therefore, it is important to revoke the modification of these molecules in time to release them from temporarily shutting down to the normal state of function. Qigong exercise can improve the transfer of local metabolism accumulative metabolites in time and simultaneously decrease oxygen consumption. A high level of blood oxygen content during Qigong exercise promotes microcirculation. This makes ischemia and anoxic organization obtain a sufficient oxygen supply in time for removing the decorations of lactylated and acetylated proteins so as to restore their normal function.

Lactate plays an essential role in proinflammatory tissue cells as a regulator. Ischemia is closely related to tissue damage, and the hypoxia caused by ischemia eventually produces a high lactate concentration. In tissue cells, during situations such as injury repair, a high lactate concentration induces the lactation of histone H3 and other molecules, which induces and promotes the expression of genes related to injury repair and transfers tissue cells from the pro-inflammatory state to the repair state [34]. This feedback function of tissue cells to the environmental response is the self-protection and repair of tissue cells. Lactate has increased the number of neuropsychiatric disorders, including depression, anxiety, and schizophrenia. Changes in lactate concentrations throughout the brain have also been found in animal models of neuropsychiatric disease [35]. This phenomenon suggests that the brain’s energy metabolism is altered in neuropsychiatric disorders, thus limiting the lactate concentration. Increasing lactate concentration will lead to lactate modification of histones and other proteins in the nucleus, resulting in changes in cell epigenetics [32]. Many patients with anxiety disorder and depression have recovered entirely through exercise. The mechanism is still unclear, but Qigong exercise may play an important role in modifying tissue and cell metabolism by reducing oxygen consumption, promoting metabolite circulation and inflammation molecules, and increasing blood oxygen content through subcellular vesicles—exosomes [36] or Caveolae-Mediated Extracellular Vesicles [37] of signaling and inflammasome functions in different cancers [38], as well as of infectious diseases, e.g., HIV [39] and SARS-CoV-2 through the production of inflammatory cytokines [40].

## 4. Perspectives of Applications: Qigong Exercise Generates Oxygen Supply and Acid-Base Balance against Hypoxic Effects: A Platform toward Preemptive Health & Medicine (PHM) to Modulate Cancer and Inflammation

The Preemptive Health & Medicine (PHM) Initiative, a concept created by Noubar Afeyan of MIT, aims to transform the present “Sick care platform”, which is expensive since it concentrates on the sickest patients at a time when interventions are costly, and outcomes are subpar [41]. As opposed to waiting for individuals to get sick, preventive health seeks to enhance, maintain, and safeguard health [42].

We suggest that the Qigong exercise, which is in line with PHM, can enhance the local hypoxic environment of tissues, encourage the circulation of metabolic accumulation throughout the body, and restore the normal metabolism of tissues and cells through calm, relaxation, and extreme breathing in the Zen tradition. The main effects are brought about by increased blood flow and oxygen supply. These include decreased oxygen consumption in brain tissues due to meditation, reduced oxygen consumption in muscle tissues due to total body relaxation, increased blood oxygen levels due to various breathing techniques, and improved microcirculation due to exercise. Changes in oxygen supply can reverse epigenetic changes by ischemia and hypoxia-induced abnormalities to glucose metabolism, lactic acid accumulation, and breastfeeding. As a result, these lactate-targeting therapies may control the Warburg effect in the tumor microenvironment (TME) to cure cancer. [43].

Humans who engage in the “vibrating preventive care” aspect of Qigong exercise, which involves regularly vibrating on acupuncture points, presumably support the “Preemptive Health & Medicine Initiative”, which advocates for lifestyle changes that encourage movement than waiting to get sick before seeking treatment. As a platform for preventive health and medicine to modify cancer, our ideas on applications include the fact that Qigong exercise provides an oxygen supply and acid-base balance against hypoxic effects, likely through lymphatic system remodeling.

A significant part of the tumor microenvironment is the lymphatic system, where lymphovascular remodeling happens in response to hypoxia and low oxygen levels, which causes metastasis and patient mortality [44]. In support of this idea, current research has broadened our understanding of lymphatics beyond their role as a “passthrough” pathway for cancer cells (CCs) to include their role as immune effectors that play a critical role in defining targeted (immune) treatment responses. Clinical aggression is determined by the anaerobic tumor metabolism driven by lactate dehydrogenase (LDHA) and the hypoxia-inducible factor HIF1alpha, as found in 175 breast cancer patients treated in a prospective study [45]. Solid tumor resistance to chemotherapy treatment forces the integration of Western medicine with Eastern medicine for their synergetic health benefits in a hypoxic microenvironment dependent on anaerobic energy metabolism for underlying specific causes. Such a combined treatment program includes dietary strategies of caloric restriction and low-carbohydrate intake to inhibit glycolysis.

In contrast, acute exercise is aimed at transiently enhancing blood flow to the tumor, thereby reducing hypoxia [46]. Aerobic exercise regulates AMPK signaling, PI3K/Akt signaling, Th1/Th2 cytokine balance related to immunity, PD-1/PD-L1 immunosuppressive signaling, and related cytokine pathways for antitumor effects [47].

A hypoxic TME heightens radioresistance; thus, disease recurrence and treatment failure continue to pose substantial challenges. However, the TME evolves under the influence of factors in systemic circulation and cellular crosstalk, underscoring its potential to be acutely and therapeutically modified. Early preclinical evidence suggests exercise may affect tumor growth, and some benefits could act to radiosensitize tumors for treatment. Intracellular perturbations in skeletal muscle reactive oxygen species (ROS) stimulate the production of numerous factors that can exert autocrine, paracrine, and endocrine effects on the prostate [48].

Cancer cells (CCs) might tilt the immunological balance or favor suppressive tumor and metastatic microenvironments through hypoxia-driven lymphatic remodeling and immunotolerance mechanisms. Specific combinatorial approaches targeting hypoxia signaling pathways might reverse this imbalance to amplify responses to targeted immunotherapies for patients with cancer [44]. For example, Hypoxia orchestrates the lymphovascular-immune ensemble in cancer [44]. Qigong exercise could safely and effectively help manage symptoms for those with, or at risk for, BCRL (breast cancer-related lymphedema) [49], primarily like through hypoxia-regulated pathways in lymphedema [50]. As such, Qigong-mediated hypoxia signaling pathways that could be utilized to reverse this imbalance and maximize the responses to targeted immunotherapies for patients with cancer is a manifestation of the mechanisms by which hypoxia-driven lymphatic remodeling and immunotolerance can be actively co-opted by CCs, tilting the immunological balance away or in favor of suppressive tumor and metastatic microenvironments.

Specifically, Qigong could help normalize the dysregulated extracellular matrix (ECM) production of hypoxic lymphatic endothelial cells (LECs), which might be partly responsible for the progression of fibrosclerosis in lymphedema. Aligned with this normalization process, Transcriptome Analysis of Hypoxic LECs reveals that in 16,000 genes expressed in LECs, 162 (1%) were up- or down-regulated by hypoxia. In comparison, 21 genes have essential functions in producing or modifying ECM [51]. More interestingly, aerobic exercise plays a protective role in primary cancer development and progression by regulating the levels of chemokines CCL2, CCL5, and their related receptors CCR2, and CCR5 in breast cancer [52]. Exercises of Qigong could establish the Breathing Signature as Vitality Score Index for holistic care through Artificial Intelligence Tools that integrate Western and Eastern medicine [4].

These hypoxic effects come from modifications of cancer-integrated tumor vasculature. Hypoxia is a characteristic of all solid tumors brought on by an imbalance in the amount of oxygen supplied to and used by the cells. Hypoxic cancer cells (CCs) trigger the complex angiogenesis process, which results in the sprouting of pre-existing capillaries to create new ones to fulfill the increasing demand for oxygen. CCs co-opt angiogenic arteries and components to develop an immunotolerant, hypoxic tumor microenvironment, resulting in treatment failure and mortality and offering oxygen for growth and an exit route for dispersion. Angiogenic factors are controlled by hypoxia-inducible factors (HIFs), the mechanistic target of rapamycin (mTOR), and the unfolded protein response (UPR): they both provide immunomodulatory and vascular actions in the tumor microenvironment. Potential treatment approaches, wherein enhancing anti-angiogenic and immunologically mediated anti-cancer responses through targeting oxygen sensing [53]. Such CCs have tight and plastic interactions with components of the tumor microenvironment (TME [54]. CCs incite the formation of new blood and lymphatic vessels from preexisting vessels to cope with their high nutrient/oxygen demand and favor tumor outgrowth. Qigong practice, acting as a novel therapeutic approach in combination with immunotherapy and chemotherapy, can highlight the significant role played by tumor-associated blood and lymphatic vasculature, not only “in thwarting immunosurveillance mechanisms and antitumor immunity” but also “in targeting the tumor vasculature and their potential to help to overcome immunotherapy resistance”. These beneficial effects might resonate with data: “mitochondria-targeted and ultrasound-responsive nanoparticles for oxygen and nitric oxide codelivery to reverse immunosuppression and enhance sonodynamic therapy for immune activation” [55]. One possible mechanism is directing hypoxic tumor microenvironment and HIF to illuminate cancer immunotherapy’s existing prospects in drug targets [56]. Future comprehensive AI-medicine-based approaches [4] might integrate multiple factors onto an index matrix to guide Qigong practice to maximize the benefits.

Conclusion: Qigong exercise generates an oxygen supply and acid-base balance against the hypoxic effects of underlying pathological conditions.

## Data Availability

Not applicable.
